# A Mechanical Treatment Method for Recycled Aggregates and Its Effect on Recycled Aggregate-Based Concrete

**DOI:** 10.3390/ma14092186

**Published:** 2021-04-24

**Authors:** Pericles Savva, Socrates Ioannou, Konstantina Oikonomopoulou, Demetris Nicolaides, Michael Frixos Petrou

**Affiliations:** 1 Latomia Pharmakas, 23 Themistokli Dervi Av., S.TA.D.Y.L. Building, P.O. Box 23504, Nicosia 1066, Cyprus; 2 Department of Civil and Environmental Engineering, University of Cyprus, 75 Kallipoleos Av., P.O. Box 20537, Nicosia 1678, Cyprus; ioannou.socrates@ucy.ac.cy (S.I.); oikonomopoulou.konstantina@ucy.ac.cy (K.O.); petrou@ucy.ac.cy (M.F.P.); 3 Frederick Research Center, P.O. Box 24729, Nicosia 1303, Cyprus; d.nicolaides@frederick.ac.cy

**Keywords:** recycled concrete aggregates, recycled aggregate concrete, adhered mortar, mineral admixes, internal curing, treatment methods, mechanical properties, durability

## Abstract

Recycle concrete aggregates (RCA) consist of natural aggregates and remnant mortar adhered to their surface. The amount, size, and morphology of the adherent remainder paste influences quality aspects of RCA, such as their bonding potential with new cement matrix in an RCA-based concrete, as well as the concrete’s overall rheological and performance characteristics. The objective of this research was to study the effect of reducing the adhered mortar in RCA, by means of a mechanical treatment method, on the performance of concrete containing RCA at different percentages. The treatment process was conducted within a concrete mixer truck drum at specific time intervals, the effect of which was determined by means of image analysis, mass loss recordings, and circularity determinations. The effect of size of treated and field RCA, as well as replacement percentages on mechanical performance and durability of high and normal strength concrete mixes, were also investigated. It was concluded that the optimal treatment duration where no further significant removal of adhered paste occurred thereon was 3 h, and concrete mixes containing 3 h treated RCA exhibited comparable performance characteristics to those of the reference concrete mix.

## 1. Introduction

The needs of the worldwide infrastructure would have never been met without concrete. Due to rapid global urbanization, driven by upturns in construction sectors in both developed and developing countries, there is indeed an exponential increase in concrete production demand, which currently reaches over 55 billion cubic meters globally [1]. With such an increasing trend follows the inevitably emerging issue of progressive depletion of the natural resources for retrieving concrete’s constituents, particularly the aggregates. Volumetrically, aggregates in concrete occupy up to 75–80% of its volume, with their global production being equally demanding. At the same time, significant amounts of construction and demolition waste are generated as a result of either existing concrete structures reaching the end of their design working life or being deemed unsafe for habitation, or due to restructuring, repairing, and maintenance plans towards urban growth.

To provide for the infrastructural needs of future generations without compromising on global natural resources, the abovementioned issue may be tackled through the utilization of recycled concrete aggregates (RCA) in concrete. This approach, in addition, would alleviate the emerging issue of construction waste disposal, especially in already congested urbanized regions. Research on RCA indeed allows for the development of effective recycling approaches, further reducing waste, as well as reducing the costs associated with disposal and storage of waste. Backed by a combination of acquired knowledge on their inherent properties, practical implementation of RCA in concreting practice may allow for natural resources to be conserved and exploited for a prolonged period of time. On the other hand, there are certain challenges that need to be addressed and tackled before adopting a large-scale utilization of RCA in the concrete industry. In particular, RCA have been known to possess certain disadvantages compared to natural aggregates, such as lower density, higher water absorption, weak adherent paste on the aggregate surface, poor mechanical properties, and durability [2,3]. In conventional concrete mixtures, the mechanical and stiffness behavior of the composite largely stems from the adherent bonding characteristics between the aggregates and the mortar, in what is currently known as the interfacial transition zone. In RCA-based concrete, however, the bonding is formed between the new mortar and the RCA, the surface of which already contains adhered mortar partially or fully covering the aggregate. In most instances, crucial information, such as the age, source origin, and mix proportions of the adhered mortar in RCA are unknown, and therefore, the material cannot be easily characterized. In addition, its surface, texture, and morphology are associated with poorer bonding characteristics, a fact that appears to yield lower mechanical properties and high variations in results. While within RCA-based concrete a second interfacial transition zone is formed (i.e., between the adhered mortar and the new mortar), the quality of the adherence within the concrete matrix has been predominantly reported as poor. Indeed, consensus suggests that mechanical properties, such as compressive strength, flexural strength, and Young’s modulus may become lower [1,4,5,6,7,8,9], and several attempts have been reported towards reducing the adhered mortar in RCA using various methods: resembled Los Angeles [4], carbonation and wrapping [2], microbial carnation precipitation [5], impregnation inside organic polymer solution and polyvinyl alcohol [6], low concentration hydrochloric acid [7,8], etc.

Kazemian et al. [7] have reported that during the evaluation of flexural strength, the loose mortar of the recycled aggregate was the most influential factor in reducing it. The introduction of 25% and 50% of RCA resulted in a reduction of 10% and 14% in flexural strength, respectively. On the contrary, identical replacement using recycled aggregates that had been subjected to mechanical treatment, led to a 3% reduction, respectively. Ismail and Ramli [10] evaluated the combination of aggregate treatment using hydrochloric acid to remove the loose adherent and calcium metasilicate to fill the remaining voids. They concluded that the treated recycled aggregates exhibited better performance than that of the non-treated recycled aggregates in terms of compressive, flexural strength, and Young’s modulus. The old mortar included micro-cracks and yielded high porosity values [11], thus it became the weakest link in RCA-based concrete. As the mortar-aggregate bond strength increased, the concrete strength was reportedly increased [4,12].

Based on the previous findings, it is therefore important to provide insights on the effect of the adhered mortar paste by understanding how its inherent quality characteristics affect the overall performance of conventionally utilized concrete. Several enhancement methods for eliminating the bound mortar have been previously suggested, such as mechanical grinding [8,13,14,15]; pre-soaking in water or acid, where lightly bound mortar was reportedly removed from the aggregate surface [16,17,18,19]; and thermal mechanical enhancement [20,21,22]. Alternative methodologies also suggested enhancement through surface treatment/crystallization [23], i.e., carbonation [24,25,26,27], calcium carbonate precipitation [9,28], and immersion in sodium silicate solution [18]. The use of the aforementioned methodologies indeed highlights useful regimes of outcomes and such techniques are comprehensively included in reviews [29] and comparisons [30]. However, concerns seem to be embedded with each methodology, as reported, such as the energy consumption throughout the experimental procedure, the cost of necessary relevant equipment and instrumentation, the scarcity of essential facilities for conducting rigorous advanced analytical techniques, as well as risks of jeopardizing the properties of the existing aggregates used in the aforementioned research. It is therefore essential, when adopting a particular methodology towards enhancement of RCA, to determine the balance between the environmental impact associated with the experimental procedure (i.e., quantitative energy consumption, embodied CO_2_ carbon footprint), the cost embedded with the usage of relevant equipment (i.e., relevant instrumentation, cost of energy associated), and the overall yielded performance.

The objective of this research is to determine, by utilizing a previously suggested mechanical treatment found in our previous work [8], whether the incorporation of treated and non-treated RCA aggregates, including non-treated RCA sand, would provide a beneficial or detrimental effect on the mechanical properties and durability of normal and high strength concrete. The reduction of the adhered mortar is achieved by means of a mechanical treatment method using a truck drum mixer as suggested in our previous work and the effect of such reduction on the performance of concrete containing RCA at different replacement percentages is studied. It is commonly accepted, on one hand, that there are advantages offered when using mechanical treatment towards reducing the adhered mortar from the aggregate, such as the elimination of the existing weakly-binding mortar and the avoidance of formation of poorly sustained ITZs within the microstructure. On the other hand, it causes a relatively adverse effect, such as increasing the circularity index of aggregates, which in turn, leads to a decrease in surface area of aggregate volume in concrete—effectively increasing the available free water (for constant w/c) in the mixture. There is a possibility that the two effects may counteract and neutralize each other, such possibility depends on the degree and extent of manifestation of each mechanism. Reducing w/c ratio may partially mitigate the circularity index increase. In any case, these issues must be taken into consideration along with the environmental impact and cost for yielding equivalent performance to that of conventionally utilized concrete. The results of our work [8] suggest that both practicality and cost effectiveness of the particular method were beneficial when considering treatment towards producing a cheaper RCA-based product to yield equivalent performance to that of conventional concrete. Therefore, the current research aims to extend upon this work by determining an optimal mechanical treatment duration where changes in RCA’s mass loss and geometry are not significant beyond a timeframe and thus the morphology, circularity, and surface area characteristics of RCA are being brought to the most optimal extent possible. In addition, its thereon aims to utilize RCA that have been treated to that optimal timeframe within concrete and to study their effect on the mechanical properties and durability of an RCA-based concrete.

Nomenclature used thereon within the text: RCA = Recycled concrete aggregates; NA = Natural Aggregates; RTC = Recycled Treated Coarse Aggregate; RFC = Recycled Field Coarse Aggregate (untreated); RFF = Recycled Field Fine Aggregate (untreated); PSD = Particle size distribution.

## 2. Materials and Methods

**Figure 1:**
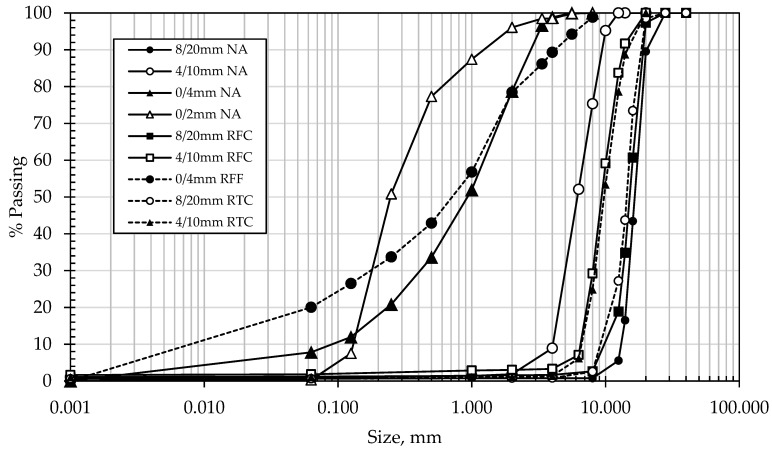
Particle size distribution of the aggregates used in the experimental.

Natural and recycled aggregates were obtained from a local quarry in Cyprus, at sizes 0/4 mm, 4/10 mm, and 8/20 mm, of which the properties were determined in accordance with the relevant EN Standards, as shown in Table 1. The particle size distribution of the aggregates is shown in Figure 1.

### 2.1. Mechanical Treatment of Aggregates

The treatment method was based on the mechanical grinding of the adhered mortar from the surface of the RCA. The aggregates were placed inside a 100 L capacity concrete drum mixer along with water, where the rotating motion allowed the aggregates to collide with each other and with the metal walls of the drum. The collision subsequently caused the material’s surface to wear, altering the geometrical and morphological characteristics by removing the loose adhered mortar. Five random samples of RCA (samples of 4/10 mm and 8/20 mm) were dried to constant mass at a temperature of 110 °C. Approximately 3 kg of each sample were selected and sieved using the 2.36 mm aperture sieve. The samples were then weighted (m_1_). Each sample was placed in the mixer drum together with water (1 part of aggregates to 1 part of water) at a speed of 0.5 rotations per second for 1, 2, 3, 4, and 5 h, respectively. The water was added to improve this method by weakening the adhered mortar. When the treatment was completed, the RCA were sieved and washed using the 2.36 mm aperture sieve. Following that, they were dried to constant mass at a temperature of 110 °C and the final mass was recorded (m_2_). The mass reduction of the RCA was calculated by using the following equation:

Residual mortar (%) = (m_1_−m_2_)/m_1,_ (1)

where:

m_1_ is the dried sample mass before treatment (g);
m_2_ is the dried sample mass after treatment (g).

This method, which essentially resembles a prolonged Los Angeles test without steel spheres, has been previously successfully used to decrease the adhered mortar to a significant degree and to discard the weaker or fractured aggregates, keeping only the stronger and sounder ones [8]. To study the effect of the treatment method in the RCA, it was necessary to focus on the geometrical and morphological characteristics of aggregates, which are directly correlated to the surface area (and thus to the water demand) and which ultimately affect the properties of fresh and hardened concrete. In this work, it was decided to study and compare the morphological characteristics of treated RCA with those of untreated RCA by means of circularity. As the alteration of circularity index of RCA was caused by the removal of the adhered mortar and possibly part of the aggregates, it was essential to examine how the circularity index is altered, solely due to the presence and amount of the adhered mortar. To achieve the particular objective, it was decided to utilize the same treatment method conducted on a large scale (i.e., 1.5 tons of aggregates mixed with an approximately equal amount of water within a rotating concrete truck drum mixer, at a rotational speed of 0.2 rps) on natural crushed aggregates (NA) studied at identical time intervals as with RCA and then determining the changes in both circularity and particle size distribution (PSD), using sieve analysis at each time interval. In this manner, deducting the difference in the rates of changes in circularity between the two cases (RCA and NA) would not only provide the removal effect of the adhered mortar on the morphological alterations of RCA, but it would also become a deciding factor towards eliminating the possibility of the authors having received RCA from a source with natural rounded aggregate embedded. It should be noted that it was not possible to establish whether part of the recycled aggregates received were naturally rounded uncrushed, due to the presence of the adhered mortar. By utilizing sieve analysis at the specific time intervals, moreover, would confirm the changes in aggregate size distribution.

To characterize the circularity of the aggregates, digital image processing was applied initially to obtain high resolution images of the samples on a digital form and then for the relevant software to perform a mathematical procedure and obtain the necessary information [31]. Both GIMP (2.10, GIMP Team, CA, USA.) and ImageJ software (1.53e, National Institutes of Health, Bethesda, MD, USA) (the former being an open-source raster graphics editor and the latter being a Java-based image processing software) were used to analyze the aggregates and determine their circularity. The process followed to determine the circularity of each aggregate consisted of initially calculating the area and the perimeter of each aggregate, using the software, and then using the following equation:

Circularity = (4 × π × A)/P ^2^ (2)

where:

A is the area of the aggregate (mm^2^);
P is the perimeter of the aggregate (mm).

Circularity values are ranging between 0.0 to 1.0 with 1.0 corresponding to a perfect circle. As the value approaches 0.0 is an indication of an increasingly elongated shape.

Samples of RCA having a mass of 3 kg were initially collected and placed on a black felt (a small portion at a time) with a measuring tape and photographed. The obtained images were then edited using GIMP removing the background and keeping the aggregates and the measuring tape as the only visible objects. Following editing, the image was analyzed using ImageJ software. Initially, the scale was set, and the image was converted into a 32-bit image. The threshold was then adjusted, and the image was analyzed for the determination of circularity. The overall circularity of each sample was taken as the average of the circularities of all the analyzed aggregate particles.

### 2.2. Concrete Properties Investigated

The current investigation included the evaluation of the concrete mixtures’ mechanical properties and durability. Test type, age of testing, and relevant standards are summarized in Table 2. For the determination of mechanical properties, compressive strength, according to EN 12390-3 [32], and splitting tensile strength, according to EN 12390-6 [33], were conducted using a CONTROLS Advantest9 hydraulic compression machine (Controls, Italy) of 5000 kN load capacity. Durability tests focused on the transport properties of concrete and material’s performance when subjected to major degradation processes. Capillary water absorption, in accordance with RILEM TC-116 [13], was conducted, where 5 mm slices were cut from 100 mm cubic samples to expose the aggregates within the cross section. The samples were maintained at 110 ± 5 °C until constant mass, cooled off to reach room temperature and sealed with an elastomer-based impermeable coating in all surfaces apart from the exposed one. The test included immersing the exposed surface at approximately 3 mm depth of 2-propanol and measuring the change in mass of the samples at specific squared time intervals. The sorptivity coefficient was then calculated based on the function slope using the equation below:

Q/(Aρ) = kt^0.5^ (3)

where:

Q is the amount of 2-propanol absorbed (g);
ρ is the density of propanol (g/mm^3^);
A is the cross-section area exposed to propanol (mm^2^);
t is the time (min);
k is the sorptivity coefficient (mm/s^0.5^).

Open porosity was determined based on BS EN 1936:2006 [34], where samples were initially oven-dried at a temperature of 65 ± 5 °C until constant mass (m_d_), placed in a vacuum desiccator, and subjected to a pressure of 2.0 ± 0.7 kPa for a duration of 2 ± 0.2 h. The desiccator was then completely filled with demineralized water and atmospheric pressure was restored. The samples were kept in the desiccator for 24 h and both immersed (m_h_) and saturated surface dry condition (m_s_) masses were recorded. The open porosity (%) was then expressed as the ratio of the volume of open pores and the apparent volume of the specimens according to the following equation:

ρ_ο_ = [(m_s_ − m_d_)/(m_s_ − m_h_)] × 100 (4)

Chloride resistance of 28-day cylindrical concrete disc specimens of 100 mm diameter and 50 mm height was determined in accordance with ASTM C-1202 using GERMANN PROOVEit apparatus system (Germann Instruments, Copenhagen, Denmark). The specimens were placed in cells containing 3% sodium chloride solution and 0.3 N sodium hydroxide solution and were subjected to a potential difference flow for 6 h. The value of the electric charge (Coulombs) was recorded during the test. Resistivity tests were conducted on 9 points on the surface of the same cylindrical samples prior to chloride resistance testing to assess these measurements. The resistivity test was carried out using a 38 mm-probe PROCEQ Resipod surface resistivity meter, which applies a current to the sample’s surface and measures the potential difference between its outer probes.

### 2.3. Mixtures and Mixture Proportions

A total of 12 mixes were prepared in accordance with the mix proportions presented in Table 3, at w/c ratios 0.25 and 0.50 for a target consistency class of S3, according to EN 206-1. To achieve the target slump range, a commercially available polycarboxylate-based superplasticizer was added during mixing and adjusted accordingly, at 1–2% by weight of cement in the concrete mix. Different mixtures were designed with RCA to NA replacement percentages at 25% and 50%. The mixture design for all mixtures was conducted as such to resemble a similar all-in aggregate grading of the total aggregate content of the reference mixture. The code names of each mix shown in Table 3 consist of the following:

- “R” for RCA.
- “F” for fine aggregates replacement (second letter in formulation nomenclatures).
- “C” for coarse aggregates replacement.
- “F” for field non-treated (third letter in formulation nomenclatures).
- “T” for treated.
- “50” or “25” represent the replacement percentage of NA.

Within this paper and throughout the discussions, the w/c ratio is suffixed at the end of each code name.

The mixtures were designed with cement contents of 400 kg/m^3^ and 864 kg/m^3^ in order to evaluate the effect of RCA in both normal and high strength concrete categories.

### 2.4. Casting and Curing

Mixes were prepared and cast using a Tecnotest AT 205 pan mixer of 150 L capacity and in accordance with BS 1881-125:2013. All types of aggregates were maintained in the laboratory, in air-dried conditions and the necessary water content corrections were made based on aggregates’ moisture content and absorption values. The samples were demoulded 24 h after casting and water-cured at a constant temperature of 22 ± 2 °C until the day of testing.

## 3. Results and Discussion

### 3.1. Circularity and Mass Loss

**Figure 2:**
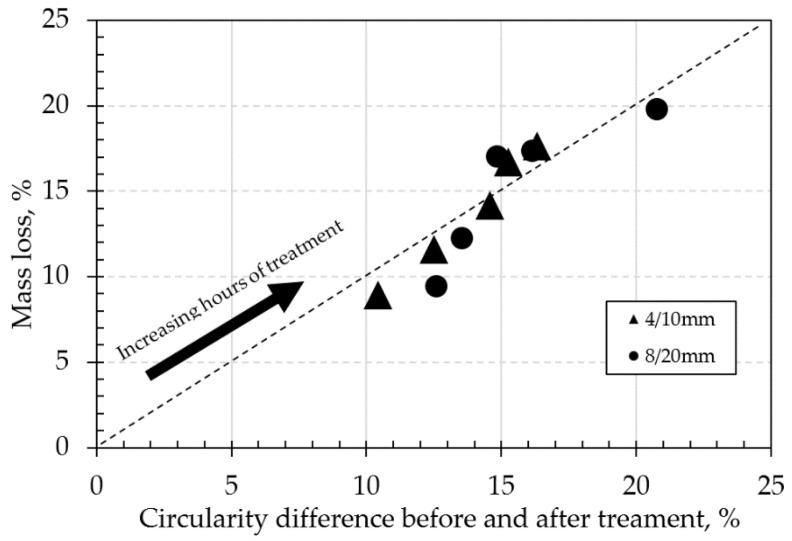
Changes is mass loss and circularity for 10 mm RCA subjected to small scale treatment for up to 5 h.

**Figure 3:**
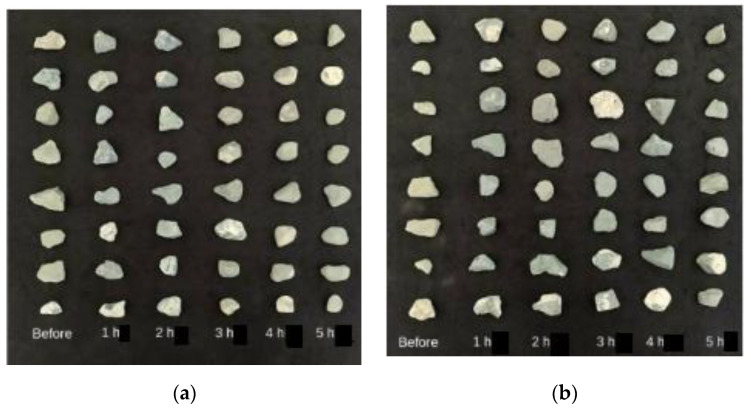
Visual changes in geometry of RCA: (**a**) 4/10 mm; (**b**) 8/20 mm subjected to mechanical treatment for up to 5 h.

**Figure 4:**
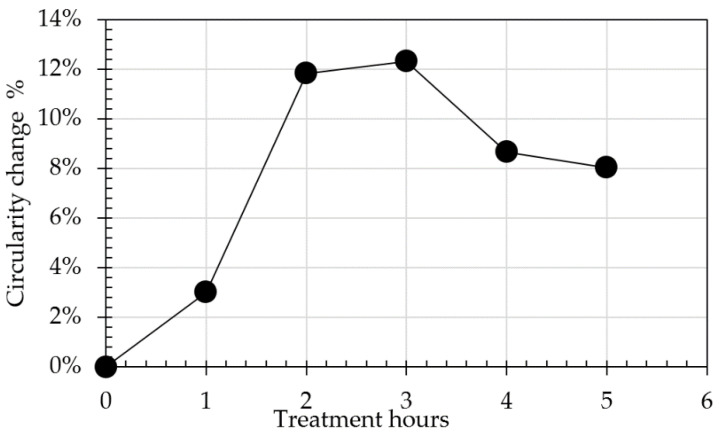
Effect of large-scale treatment hours on the particle size distribution of natural crushed aggregates (NA) 8/20 mm.

**Figure 5:**
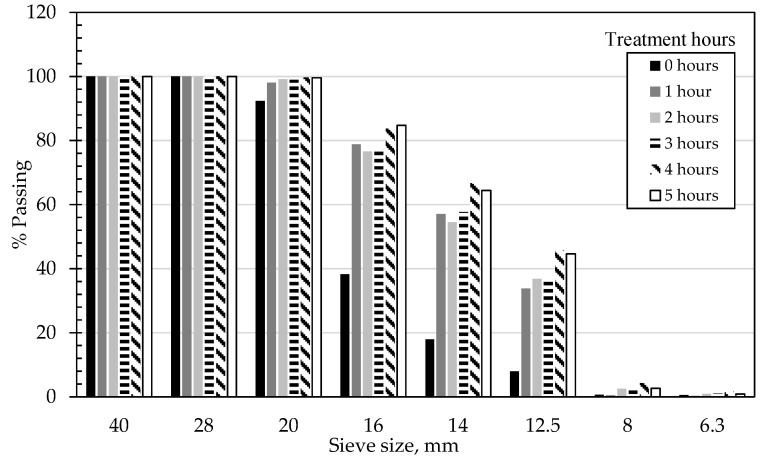
Effect of treatment hours on the particle size distribution of 8/20 mm NA.

**Figure 6:**
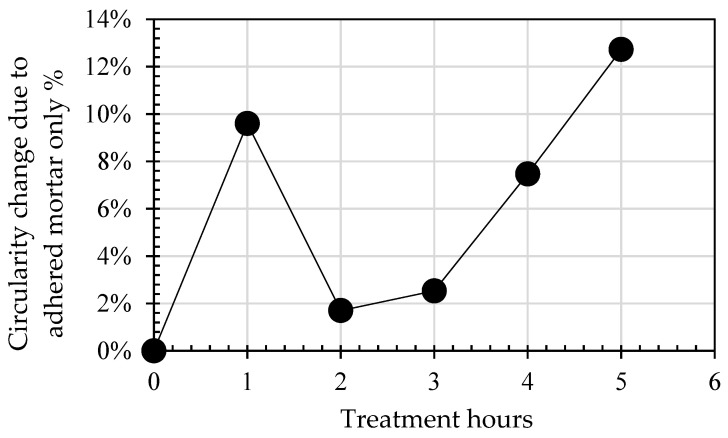
Effect of treatment hours on the difference between circularity changes of RCA and NA 8/20 mm.

Changes in mass loss and circularity for both 4/10 mm and 8/20 mm RCA and NA, along with the effect of the presence of adhered mortar on the changes in circularity across the treatment hours, are shown in Table 4 and in Figure 2, Figure 3, Figure 4, Figure 5 and Figure 6. In general, the results showed that the mechanical treatment up to 5 h reduced both 4/10 mm and 8/20 mm RCA aggregates’ mass down to 18% and 20% of their initial values, respectively, and increased their circularity up to almost 17% and 21%, respectively. During the first 3 h of treatment, it was evident that 4/10 mm aggregates suffered a steady mass loss rate of approximately 2% for each hour of treatment—a trend which was also apparent on the circularity readings for the same duration and on an almost linear rate. For the 8/20 mm, however, mass loss was more intense within the first 3 h compared to 4/10 mm. Thereafter, mass loss was still occurring at comparable lower rates in both cases. However, circularity variations were not as significant, apart from the case of 8/20 mm RCA, where a sharp increase to almost 21% occurred on the 5th hour of treatment. These results suggest that the optimal duration, in terms of cost and performance efficiency, would be approximately 3 h. Although a noticeable effect was observed on 8/20 mm RCA treated up to 5 h, the additional power consumption needed for this task renders the mechanical treatment of aggregates for such a long period as an economically unviable option. From this perspective, for the large-scale experiments, it was decided that RCA would receive a 3 h treatment. The results presented in Table 4 showed a higher circularity difference from RCA to treated aggregates after 3 h, which is attributed to the larger mass of aggregates (1.5 tons) added in the concrete mixer drum. In the case of treatment applied to NA 8/20 mm aggregates as shown in Figure 5, the results suggest that the differences in circularity were not as significant beyond a 3 h treatment duration as in the case of the first 2 h. A “crushing” effect appeared to have occurred past the 3 h timeline, which is also confirmed by the sieve analysis in Figure 6, where the percentage passing at smaller sieve sizes (i.e., from 16 mm to 6.3 mm sieves) significantly increased on the 4th and 5th hours by almost 10%. The particular results also indicate that with the increase of higher amounts of fines passing through the smaller sieves beyond the 3rd hour, there was indeed mass loss occurrence and circularity alterations, as evident in Figure 5. When deducting the circularity changes of RCA 8/20 mm from those of NA 8/20 mm at equal treatment time intervals, the results in Figure 6 showed that the sole effect of the adhered mortar itself was more drastic on the morphology alterations predominantly during the first hour. Upon reaching the second hour, variations dropped to a minimum (i.e., less than 1.75%) and were similarly maintained at such low regimes during the 3rd hour as well. The variations, however, were again increased sharply at the 4th and 5th hours of treatment, a behavior which probably confirms the “crushing” effect observed in Figure 5. Overall, the results suggest that there was indeed an effect caused by the sole presence of the adhered mortar on the aggregates, which led to geometrical alterations across the examined time intervals of treatment.

### 3.2. Mechanical Properties

**Figure 7:**
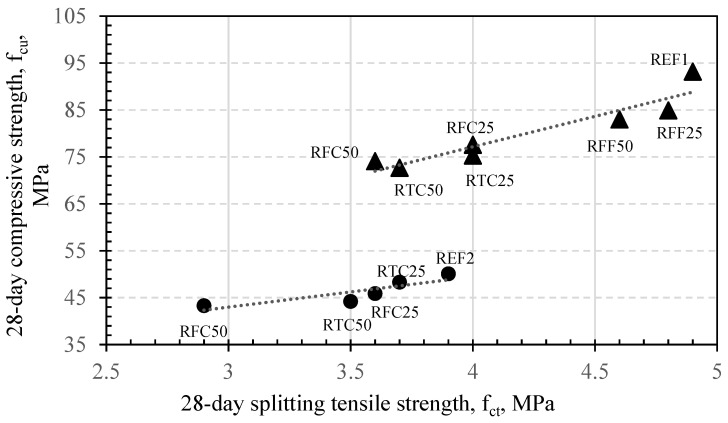
Relationship between f_ct_ and f_cu_ of concretes at 28 days ▲ = w/c ratio 0.25; ● = w/c ratio 0.5.

Table 5 and Figure 7 show the results of the compressive and splitting tensile strength development, for all mixtures at 7 and 28 days. Compressive strength is an indicator of the evolution of the microstructural density, mainly of the cement paste, whereas split tensile strength provides an indirect measure of the bonding adherence between the cement matrix and the aggregate. All concrete mixes exhibited an increase in compressive strengths from 7 days to 28 days, apart from the case of RTC50 (0.25), whose 7 and 28 day values were identical. The highest 28 day f_cu_ value was observed in REF1 and the highest early strengths were exhibited by RFF25 (0.25), RTC25 (0.25), and RTC50 (0.25), reaching up to 96% of their 28 day strength values at 7 days. For low w/c ratios, higher replacement contents of coarse recycled aggregates, either treated or untreated, reduced both compressive and splitting tensile strengths at 28 days, although the compressive to splitting tensile strength ratio for each of those combinations appeared to be maintained relatively constant. This is apparent in Figure 7 when observing the slopes of the trend lines, which were depicted within a good linear regression. When incorporating, however, recycled fine aggregates in low w/c ratio concretes in either low or high amounts, there seemed to be a reduction in compressive strengths; this may be possibly attributed to the existence of unhydrated amounts of cement clinker within the microstructure of the concretes, due to the high cement contents. Splitting tensile values of these combinations were, however, relatively similar to those of the REF.

**Figure 8:**
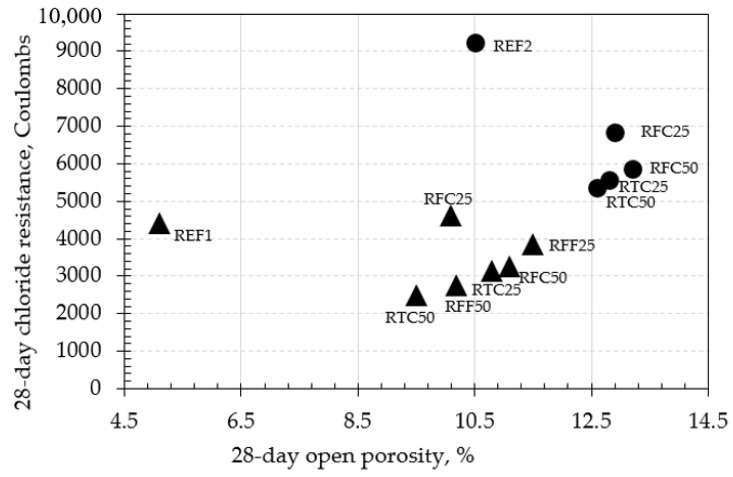
Relationship between open porosity and chloride resistance of concretes at 28 days (▲ = w/c ratio 0.25; ● = w/c ratio 0.5).

When considering the w/c ratio of 0.5, it can be seen that there is a good and almost linear rate of reduction in both compressive and splitting tensile strengths when 25% of either treated or untreated aggregates were incorporated. In this case, RTC25 exhibited slightly improved mechanical properties compared to that of RFC25. This behavior may be underpinned by the fact that treatment of aggregates contributed to a slightly improved microstructure compared to utilization of field aggregates at the same contents, in agreement with the results of the open porosity values of the same concretes, shown in Figure 8. An interesting behavior observed was that of RFC50 (0.5) and RTC50 (0.5) both yielded almost identical 28 day compressive strengths. This denoted that aggregate treatment may not affect the early-age compressive strengths of high w/c ratio concretes when RCA are present in high amounts. A possible explanation for this observation would be an early formation of secondary ITZs, characterized by an increased thickness and which adversely affected the microstructural properties of the concrete. There was, however, a considerable difference (i.e., almost 40%) between the splitting tensile strengths of the two abovementioned combinations. The results suggest that the effect of mechanical treatment of recycled aggregates on the bonding characteristics of concretes may be more beneficial to mixes of higher w/c ratios—an observation that also coheres with previous research [8]. In low w/c ratios, this effect was still apparent but to a lesser extent. It may, therefore, indicate a possibility that the effect of geometrically altered aggregates in concrete could be somewhat hindered by the significantly high cement contents in the mixes. Nevertheless, mechanical treatment still appeared to positively affect the early strengths of concretes at low w/c ratios.

It was also observed that RFC50 concrete exhibited the lowest 28 day splitting tensile strengths compared to the reference (up to 22% reduction), regardless of the w/c ratio. These reductions indicated that the adherence between the untreated RFC and the new mortar matrix was most probably poorly sustained. It can be therefore suggested that the presence of remnant mortar on the surfaces of untreated RFC, when this is present in high amounts within the mixes, negatively affects the bonding characteristics of the concrete in the interfacial transition zone.

### 3.3. Durability Properties

Open porosity, sorptivity, chloride resistance, and resistivity results of the investigated concrete mixtures at 1, 3, 7, and 28 days are summarized in Table 6. Such behavior may be attributed to a number of probable mechanisms, which may have been acting either independently or in a synergistic manner, such as the rapid formation of a shell during cement hydration due to high cement fineness, the formation of a primary and a secondary ITZ due to the presence of recycled concrete aggregates, and the internal curing mechanism activated in mixtures containing recycled aggregates. The study of the predominant mechanism(s) controlling the specific experimental observation will be the matter of further investigation by the authors’ team.

**Figure 9:**
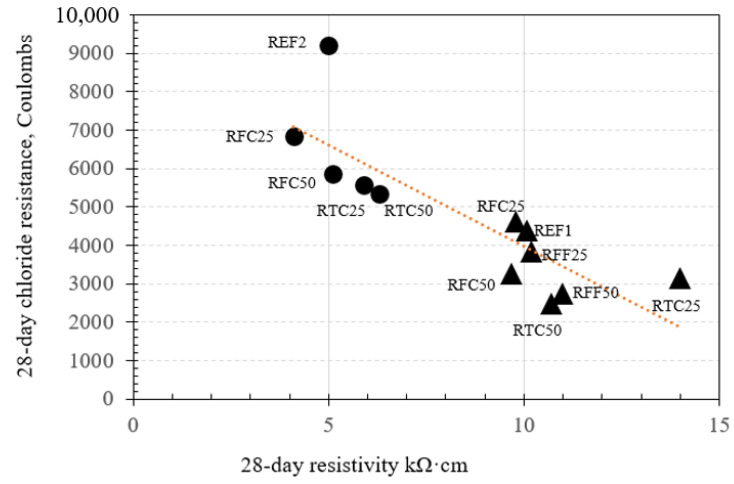
Relationship between rapid chloride resistance and resistivity of concretes at 28 days (▲ = w/c ratio 0.25; ● = w/c ratio 0.5).

**Figure 10:**
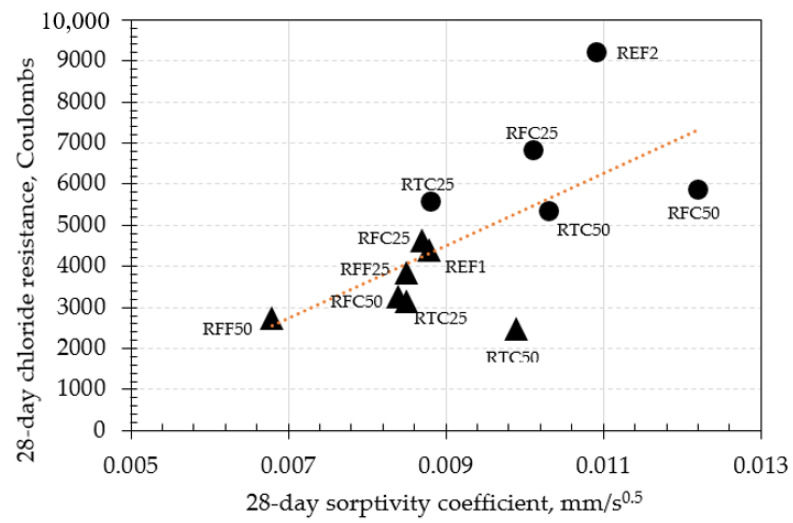
Relationship between rapid chloride penetration resistance and capillary water absorption of concretes at 28 days (▲ = w/c ratio 0.25; ● = w/c ratio 0.5).

The relationships between the relevant durability properties of mixtures at 28 days are shown in Figure 8, Figure 9 and Figure 10.

It was observed that the microstructural development of almost all the investigated mixtures followed a reasonable trend, i.e., pore volumes and permeabilities were mainly higher at day 1, 3, and 7 days, while being reduced towards the age of 28 days. This behavior was evident apart from the cases of RFF50 (0.25). Based on the results shown in Figure 8, Figure 9 and Figure 10 the concrete mixes with w/c ratio of 0.50 exhibited higher porosity, sorptivity, as well as lower resistivity than the corresponding values of the mixtures with w/c ratio of 0.25. When RTC was incorporated at 50% replacement by mass (RTC50), there appeared to be a reduction in the porosity of concrete mixtures with 0.25 w/c ratio of approximately 30%, compared to that of reference, as well as an improvement in the chloride resistance by almost 35%. The same behavior was also observed when considering incremental replacements of fine RCA within concretes at 0.25 w/c ratio. There was no clear correlation, however, when RFC was incorporated at 25% and 50% percentages (RFC25 and RFC50) at the same w/c ratio. The particular results of chloride resistance are also validated via the resistivity tests in Figure 9, where the incorporation of RCA led to mixes with higher resistivity values, with the exception of RTC25 (0.25) and RTC50 (0.25). Although RTC50 (0.25) was associated with an approximately 25% lower chloride resistance than that of RTC25 (0.25), it surprisingly gave lower resistivity values than expected. Another interesting aspect was observed when either high or low amounts of coarse recycled aggregates were incorporated in concrete (i.e., RFC25 and RFC50), the resistivity remained unaffected regardless of the Coulomb values yielded for each mix.

The relationship between the chloride resistance and capillary water absorption coefficient of mixes at 28 days, is shown in Figure 10. Both of these properties are permeation-based, and they directly relate to the degree of interconnectivity of the capillary pore network of concrete. The results showed that the mechanical treatment contributed to the reduction of permeability of concretes when comparing equivalent replacement contents (i.e., treated and untreated RCA) and at both w/c ratios investigated. It was observed that, in concrete mixtures of low w/c ratio, the incorporation of either treated, untreated, fine, or coarse RCA, specifically at 25% by mass, did not affect the sorptivity coefficients. Such behavior was apparent regardless of the corresponding chloride resistance for each mix. In particular, all RFC25, RFF25, and RTC25 mixes had almost identical sorptivity coefficients, while RTC25 exhibited the highest chloride resistance between these.

An opposing behavior was observed when the incorporated amounts of either fine or coarse RCA (treated or untreated) in low w/c concrete mixtures was 50%, instead of 25% by mass. The 3 hs mechanical treatment of RTC led to an increase in the sorptivity coefficients of RTC50 concrete mixture by almost 20% compared to REF, although it maintained the chloride resistance at a comparable level to that of RFC50.

It was observed that in concretes of w/c ratio 0.50, the incremental addition of 3 h mechanically treated aggregates at 25% and 50% by mass, reduced the sorptivity coefficients at a linear rate (approximately a 0.0011 mm/s^−0.5^ difference for each increment). These changes were more drastic than those that occurred in concretes of low w/c ratios. Such reduction indicates a beneficial effect that the mechanical treatment offered, which empowered the pore network of the investigated concretes. This was also reflected in the compressive strength development of the aforementioned concretes presented in Table 5.

An interesting behavior in chloride resistance, as seen from the results in Figure 8, was observed by REF1 and REF2 where both concretes exhibited values not fully corresponding to their equivalent open porosity. Particularly, in the case of REF1, the porosity value was significantly lower than the other mixtures even by 50% in some cases, whereas its RCPT value did not seem to justify such pore network—recorded as the second highest of all, at a value of almost 4400 Coulombs and similar to that of RFC25 (0.25). The same behavior was observed for REF 2, where the RCPT value appeared to be the highest of all, surpassing the rest of mixtures by a significant difference (25% higher than the second highest value, i.e., that of RFC25 (0.5)). Its porosity value, however, was approximately 20% lower than the second lowest (i.e., RTC50 (0.5)). Such behavior may be attributed to a number of probable mechanisms, which may have been acting either independently or in a synergistic manner, such as the rapid formation of a shell during cement hydration due to high cement fineness, the formation of a primary and a secondary ITZ due to the presence of recycled concrete aggregates, and the internal curing mechanism activated in mixtures containing recycled aggregates. The study of the predominant mechanism(s) controlling the specific experimental observation will be the matter of further investigation by the authors’ team.

## 4. Conclusions

An optimal treatment duration for recycled concrete aggregates was determined based on the observations in mass loss and geometrical alterations of recycled aggregates. Following the treatment process, the aggregates were utilized in concrete combinations at w/c ratios 0.50 and 0.25, aiming to study their effect on the mechanical properties and durability of concrete with natural crushed and recycled aggregates, at strategic increments in the mix design. The following conclusions were drawn:

- An optimal duration for mechanical treatment of RCA (beyond that, no significant changes in circularity and mass loss of RCA were observed) was found to be 3 h. This was further evidenced through a large-scale treatment process that yielded comparable circularity differences.
- At low w/c ratios, higher replacement contents of coarse RCA, either treated or untreated, reduced both compressive and tensile strengths of concrete mixtures at 28 days. When incorporating recycled fine aggregates, and particularly at a replacement content of 25% by mass, the compressive and tensile strengths of concrete mixtures were the closest to those of the reference.
- Durability properties seem to be slightly affected by the addition of recycled aggregates. A 3 h treatment on aggregates, which were incorporated at high replacement percentages within low w/c ratio mixes, led to an increase in the sorptivity coefficients by almost 20%, although it maintained the chloride resistance at comparable levels to those of concretes containing untreated aggregates at high amounts.
- It was observed that in concretes of w/c ratio 0.50, the incremental addition of 3 h mechanically treated aggregates at 25% and 50% by mass reduced the sorptivity coefficients at a linear rate (approximately a 0.0011 mm/s^−0.5^ difference for each increment). These changes were more drastic than those that occurred in concretes of low w/c ratios. Such reduction indicates a beneficial effect due to the mechanical treatment which enhanced the pore network of the investigated concretes.

## Figures and Tables

**Table 1:** Properties of aggregates.

Properties	Relevant Standard	4/10 mm NA ^1^	4/10 mm RTC ^2^	4/10 mm RFC ^3^	0/4 mm NA	0/4 mm RFF ^4^	0/2 mm NA	8/20 mm RTC	8/20 mm RFC	8/20 mm NA
Los Angeles coefficient (LA) (%)	ASTM C131	29	15	32	-	-	-	15 *	32 *	29 *
Particle Density (Kg/m^3^)	EN 1097-6	2473	2430	2517	2267	2299	2530	2400	2430	2500
Particle Density, SSD (Kg/m^3^)	EN 1097-6	2567	2539	2681	2378	2413	2580	2490	2530	2600
Water Absorption (%) (WA) (%)	EN 1097-6	3.79	4.48	6.52	4.89	4.95	1.8	4.0	4.40	4.10
Soundness (%)	ASTM C88	30	14	41	-	-	-	14 *	41 *	30 *
Flakiness Index	EN 933-3	16	4	5	-	-	-	6	5	7
Shape Index	EN 933-4	9	5	7	-	-	-	15	16	9

^1^ NA = Natural Aggregates. ^2^ RTC = Recycled Treated Coarse Aggregate.^3^ RFC = Recycled Field Coarse Aggregate (untreated). ^4^ RFF = Recycled Field Fine Aggregate (untreated). * Values from Dimitriou et al. [8].

**Table 2:** Experiments, age of testing, and standards.

Test			Standard	Age of Testing (Days)	Specimens
Hardened Concrete	Mechanical	Compressive Strength	EN 12390-3 [32]	1, 3, 7, 28	3 cubes
		Tensile Splitting Strength	EN 12390-6 [33]	7, 28	2 cylinders
	Durability	Capillary Water Absorption	Ref. [2]	1, 3, 7, 28	3 cubes
		Open Porosity	Ref. [2]	1, 3, 7, 28	3 cubes
		Chloride Ion Resistivity	ASTM C-1202	28	3 cylinders

**Table 3:** Mix proportions, nomenclature, and replacement percentages of RCA in concrete mixes.

		Water	Portland Cement	Sand 0/2 mm	Sand 0/4 mm	Aggregate 4/10 mm	Aggregate 8/20 mm	Recycled Sand 0/4 mm	Recycled Aggregate 4/10 mm	Recycled Aggregate 4/10 mm	Recycled Aggregate 8/20 mm	Recycled Aggregate 8/20 mm
	Code	-	PC	-	-	NA 4/10 mm	NA 8/20 mm	RFF 0/4 mm	RFC 4/10 mm	RTC 4/10 mm	RFC 8/20 mm	RTC 8/20 mm
w/c Ratio	Description	-	CEM I 52.5N to EN197-1	Natural Fine Sand	Natural Diabase Sand	Natural Crushed Aggregate	Natural Crushed Aggregate	Field Sand (Untreated)	Field Aggregate (Untreated)	Mechanically Treated Aggregate	Field Aggregate (Untreated)	Mechanically Treated Aggregate
		Constituent Contents in Mix Design (kg/m^3^)										
0.25	REF1	216	864	184	335	730	-	-	-	-	-	-
	RFF25	216	864	183	251	730	-	84	-	-	-	-
	RFF50	216	864	184	167	730	-	167	-	-	-	-
	RFC25	216	864	183	334	548	-	-	183	-	-	-
	RFC50	216	864	184	334	365	-	-	365	-	-	-
	RTC25	216	864	184	335	547	-	-	-	182	-	-
	RTC50	216	864	184	335	365	-	-	-	365	-	-
0.50	REF2	200	400	406	266	386	629	-	-	-	-	-
	RFC25	200	400	407	266	289	471	-	96	-	157	-
	RFC50	200	400	407	266	192	314	-	192	-	314	-
	RTC25	200	400	406	266	289	471	-	-	96	-	157
	RTC50	200	400	406	266	193	314	-	-	193	-	314

**Table 4:** Changes in geometry and mass of RCA subjected up to small-scale and large-scale treatment.

**RCA 4/10 mm**									
**Hours of Mechanical Treatment**	**Circularity Recordings** **(Small Scale)**			**Mass Loss Recordings (Kg)**			**Circularity Recordings** **(Large Scale)**		
	**Before Treatment**	**After Treatment**	**% Variation**	**Before Treatment**	**After Treatment**	**% Variation**	**Before Treatment**	**After Treatment**	**% Variation**
1	0.6735	0.7522	10.45	2.971	2.706	8.91	-	-	-
2	0.6667	0.7622	12.53	2.884	2.549	11.60	-	-	-
3	0.6523	0.7637	14.58	2.951	2.534	14.15	0.6605	0.7938	16.80
4	0.6561	0.7745	15.28	2.969	2.472	16.71	-	-	-
5	0.6534	0.7811	16.35	2.980	2.455	17.62	-	-	-
**RCA 8/20 mm**									
**Hours of Mechanical Treatment**	**Circularity Recordings** **(Small Scale)**			**Mass Loss Recordings (Kg)**			**Circularity Recordings** **(Large Scale)**		
	**Before Treatment**	**After Treatment**	**% Variation**	**Before Treatment**	**After Treatment**	**% Variation**	**Before Treatment**	**After Treatment**	**% Variation**
1	0.6559	0.7505	12.60	2.995	2.711	9.47	-	-	-
2	0.6570	0.7598	13.53	2.996	2.629	12.25	-	-	-
3	0.6492	0.7623	14.84	2.980	2.472	17.04	0.6516	0.7864	17.20
4	0.6469	0.7713	16.13	2.996	2.476	17.35	-	-	-
5	0.6220	0.7850	20.76	2.994	2.401	19.79	-	-	-

**Table 5:** Compressive and splitting tensile strength of concrete mixtures.

w/c Ratio	Mixture	*f_em._* (MPa)				*f_ct_* (MPa)	
		1 Day	3 Days	7 Days	28 Days	7 Days	28 Days
0.25	REF1	71.1	75.3	86.8	93.2	4.3	4.9
0.25	RFF25	64.6	67.8	76.9	84.9	3.7	4.8
0.25	RFF50	68.8	70.7	73.4	83.0	4.0	4.6
0.25	RFC25	61.9	62.8	68.5	77.6	3.9	4.0
0.25	RFC50	62.7	64.5	66.7	74.1	3.8	3.6
0.25	RTC25	69.2	70.9	71.9	75.3	3.9	4.0
0.25	RTC50	62.0	69.9	69.9	72.7	3.6	3.7
0.5	REF2	35.6	40.5	45.1	50.1	3.7	3.9
0.5	RFC25	29.3	36.3	39.9	45.9	3.1	3.6
0.5	RFC50	24.9	32.1	36.1	43.3	2.6	2.9
0.5	RTC25	31.3	34.0	41.2	48.3	3.1	3.7
0.5	RTC50	28.2	32.6	38.1	44.2	3.0	3.5

**Table 6:** Durability properties of the investigated combinations.

		Porosity (%)				Sorptivity (mm/√s)				RCP (Coulombs)	Resistivity
w/c Ratio	Mix	1 Day	3 Days	7 Days	28 Days	1 Day	3 Days	7 Days	28 Days	28 Days	28 Days
0.25	REF1	8.2	6.1	5.7	5.1	0.0071	0.0126	0.0103	0.0088	4392	10.1
0.25	RFF25	12.5	13.4	12.8	11.5	0.0087	0.0089	0.0090	0.0085	3838	10.2
0.25	RFF50	9.7	9.5	10.9	10.2	0.0093	0.0073	0.0067	0.0068	2737	11
0.25	RFC25	12.5	12.8	10.6	10.1	0.0089	0.0089	0.0089	0.0087	4619	9.8
0.25	RFC50	13.1	13.7	11.9	11.1	0.0118	0.0108	0.0100	0.0084	3240	9.7
0.25	RTC25	9.8	9.4	11.4	10.8	0.0098	0.0096	0.0085	0.0085	3137	14
0.25	RTC50	11.1	9	10.9	9.5	0.013	0.0103	0.0098	0.0099	2469	10.7
0.5	REF2	13.8	12.7	11.7	10.5	0.0135	0.0137	0.0093	0.0109	9222 *	5
0.5	RFC25	19.3	17.5	14.5	12.9	0.0126	0.0114	0.0106	0.0101	6840	4.1
0.5	RFC50	19.4	17.8	14.9	13.2	0.0146	0.0131	0.0127	0.0122	5873	5.1
0.5	RTC25	19	16.6	14.4	12.8	0.0125	0.0121	0.0116	0.0088	5579	5.9
0.5	RTC50	17.6	14.7	14.6	12.6	0.0155	0.0140	0.0135	0.0103	5350	6.3

* The particular value was a predicted one by the instrumentation as of the overflow issues caused during sample testing.

## Data Availability

The data presented in this study are available on request from the corresponding author. The data are not publicly available due to privacy restrictions.
